# Menstrual Hygiene: How Hygienic is the Adolescent Girl?

**DOI:** 10.4103/0970-0218.40872

**Published:** 2008-04

**Authors:** A Dasgupta, M Sarkar

**Affiliations:** Department of Preventive and Social Medicine, All India Institute of Hygiene & Public Health, Kolkata, West Bengal, India

**Keywords:** Adolescent girl, menstrual hygiene, reproductive tract infections, RTI, sanitary pad

## Abstract

**Background:**

Menstruation and menstrual practices are still clouded by taboos and socio-cultural restrictions resulting in adolescent girls remaining ignorant of the scientific facts and hygienic health practices, which sometimes result into adverse health outcomes.

**Objectives:**

(i) To elicit the beliefs, conception and source of information regarding menstruation among the study population and (ii) to find out the status of menstrual hygiene among adolescent girls.

**Materials and Methods:**

A descriptive, cross-sectional study was conducted among 160 adolescent girls of a secondary school situated in the field practice area of Rural Health Unit and Training Center, Singur, West Bengal, with the help of a pre-designed and pre-tested questionnaire. Data were analyzed statistically by simple proportions.

**Results:**

Out of 160 respondents, 108 (67.5%) girls were aware about menstruation prior to attainment of menarche. Mother was the first informant regarding menstruation in case of 60 (37.5%) girls. One hundred and thirty-eight (86.25%) girls believed it as a physiological process. Seventy-eight (48.75%) girls knew the use of sanitary pad during menstruation. Regarding practices, only 18 (11.25%) girls used sanitary pads during menstruation. For cleaning purpose, 156 (97.5%) girls used both soap and water. Regarding restrictions practiced, 136 (85%) girls practised different restrictions during menstruation.

**Conclusions:**

Menstrual hygiene, a very important risk factor for reproductive tract infections, is a vital aspect of health education for adolescent girls. Educational television programmes, trained school nurses/health personnel, motivated school teachers and knowledgeable parents can play a very important role in transmitting the vital message of correct menstrual hygiene to the adolescent girl of today.

Menstruation is a phenomenon unique to the females. The onset of menstruation is one of the most important changes occurring among the girls during the adolescent years. The first menstruation (menarche) occurs between 11 and 15 years with a mean of 13 years.

Adolescent girls constitute a vulnerable group, particularly in India where female child is neglected one. Menstruation is still regarded as something unclean or dirty in Indian society. The reaction to menstruation depends upon awareness and knowledge about the subject. The manner in which a girl learns about menstruation and its associated changes may have an impact on her response to the event of menarche. Although menstruation is a natural process, it is linked with several misconceptions and practices, which sometimes result into adverse health outcomes.

Hygiene-related practices of women during menstruation are of considerable importance, as it has a health impact in terms of increased vulnerability to reproductive tract infections (RTI). The interplay of socio-economic status, menstrual hygiene practices and RTI are noticeable. Today millions of women are sufferers of RTI and its complications and often the infection is transmitted to the offspring of the pregnant mother.

Women having better knowledge regarding menstrual hygiene and safe practices are less vulnerable to RTI and its consequences. Therefore, increased knowledge about menstruation right from childhood may escalate safe practices and may help in mitigating the suffering of millions of women.

With the above background, this study was undertaken with the following objectives:

1. To elicit the beliefs, conception and source of information regarding menstruation among the study population.
2. To find out the status of menstrual hygiene among adolescent girls.

## Materials and Methods

*Type of study*: Community-based cross-sectional observational study.

*Place of study*: The present study was undertaken among the adolescent schoolgirls in the field practice area of Rural Health Unit and Training Center, Singur, Hooghly district, West Bengal. A secondary school “Gobindapur Purnachandra Vidyayatan” situated at Dearah, Singur block was selected for this study.

*Duration of study*: One month, 15.12.06-15.1.07.

*Study population*: One hundred and sixty girls from the above-mentioned secondary school of one class (class IX) were selected. It had four sections and one section was covered every week.

*Study tool*: A pre-designed, pre-tested questionnaire.

*Methodology*: After taking permission from the school authority, the class teachers of the four sections of class IX were explained the purpose of the study and rapport was built up with the girl students and verbal consent was obtained from them. Briefing was done to the students regarding the questionnaire provided to them. This pre-designed, pre-tested and structured questionnaire included topics relating to awareness about menstruation, source of information regarding menstruation, hygiene practiced during menstruation and restricted activities practiced during menstruation. At the end of the study, after collection of the questionnaire from the students, all their queries were answered satisfactorily by the research worker.

*Statistical analysis*: Data obtained were collated and analyzed statistically by simple proportions.

## Results

This study shows that the age of menstruating girls ranged from 14 to 17 years, maximum (76.25%) number of girls being between 14 and 15 years of age group. Among 160 respondents in the present study, 152 (95%) were Hindus, whereas only eight (5%) girls were Muslims. Fathers of most of the girls were farmers (45%), followed by businessmen (26.25%), service holders (12.5%) and daily wage labourers (8.75%). Mothers of most of the respondents were housewives (93.75%).

Table 1 shows that 108 (67.5%) girls were aware about menstruation prior to attainment of menarche. Among 160 respondents, mother was the first informant only in case of 60 (37.5%) girls. Other sources of information were friends and relatives in case of 46 (28.75%) girls and two (1.25%) girls, respectively. In the present study, the mean age of menarche of the respondents was 12.8 years.

**Table 1:** Information about menarche (*n* = 160)

Information	No.	Percentage
Age of menarche (years)		
10	2	1.25
11	8	5
12	50	31.25
13	66	41.25
14	24	15
15	10	6.25
Awareness about menstruation during menarche	108	67.5
Source of Information before menarche		
Mother	60	37.5
Relative	2	1.25
Friend	46	28.75
Nil	52	32.5

Table 2 shows the different beliefs and conception about menstruation among the respondents. It was observed that 138 (86.25%) girls believed it as a physiological process. Ten (6.25%) girls believed it as a curse of God, eight (5%) girls believed that it was a disease and four (2.5%) girls believed it be result of some sin. Most of the girls (97.5%) did not know about the source of menstrual bleeding. More than half of the girls (51.25%) were ignorant about the use of sanitary pads during menstruation.

**Table 2:** Perception about menstruation (*n* = 160)

Beliefs/conception	No.	Percentage
What is the cause of menstruation?		
It is a physiological process	138	86.25
It is a curse of God	10	6.25
It is caused by a sin	4	2.5
It is caused by a disease	8	5
From which organ does the menstrual blood come?		
Uterus	4	2.5
Don't know	156	97.5
What absorbent should be ideally used during menstruation?		
Sanitary pad	78	48.75
Cloth piece	82	51.25

Table 3 depicting the practices during menstruation shows that 18 (11.25%) girls used sanitary pads during menstruation, 68 (42.5%) girls used old cloth pieces and 10 (6.25%) girls used new cloth pieces. Sixty-four (40%) girls used both cloth pieces and sanitary pads during menstruation. Cleanliness of external genitalia was unsatisfactory (frequency of cleaning of external genitalia is 0-1/day) in case of 24 (15%) girls. For cleaning purpose, 156 (97.5%) girls used both soap and water. More than half of the respondents (51.25%) did not possess a covered toilet. Regarding the method of disposal of the used material, 118 (73.75%) girls reused cloth pieces and 92 (57.5%) girls properly disposed the cloth pieces or sanitary pads used, i.e. they wrap the used cloth piece or sanitary pad in a paper bag and disposed in a place used for solid waste disposal.

**Table 3:** Practice of menstrual hygiene (*n* = 160)

Menstrual hygiene	No.	Percentage
Use of material during menstruation		
Sanitary pads	18	11.25
New cloth pieces	10	6.25
Old cloth pieces	68	42.5
All the above	64	40
Cleaning of external genitalia		
Satisfactory*	136	85
Unsatisfactory†	24	15
Use for cleaning purpose		
Only water	4	2.5
Soap and water	156	97.5
Maintenance of privacy		
Yes	78	48.75
No	82	51.25
Method of disposal‡		
Cloth pieces reused	118	73.75
Sanitary pads/cloth pieces	92	57.5
disposed properly		

* Satisfactory: Frequency of cleaning of external genitalia is ≥2/day;

† Unsatisfactory: Frequency of cleaning of external genitalia is 0-1/day;

‡ Multiple response

Regarding different types of restrictions practiced during menstruation [Table 4], only 24 (15%) girls did not practice any restriction. One hundred and thirty-six (85%) girls practiced different restrictions during menstruation. Among them, 96 (70.59%) girls did not attend any religious occasion, 68 (50%) girls did not eat certain foods such as sour foods, banana, radish and palm. Fifty-eight (42.65%) girls did not play, 46 (33.82%) girls did not perform any household work, 22 (16.18%) girls did not attend school and 14 (10.29%) girls did not attend any marriage ceremony during the menstrual period.

**Table 4:** Restrictions practiced during menstruation (*n* = 160)

Restrictions	No.	Percentage
Not practiced	24	15
Practiced for*	136	85 (100)
Any religious occasion	96	70.59
Marriage	14	10.29
School	22	16.18
Playing	58	42.65
Household work	46	33.82
Certain foods	68	50

* Multiple response

## Discussion

This study shows that the age of menstruating girls ranged from 14 to 17 years with maximum number of girls between 14 and 15 years of age. Similar study conducted by Deo *et al*.(1) reported that the age of menstruating girls ranged from 12 to 17 years with maximum number of girls between 13 and 15 years of age. In the present study, the mean age of menarche of the respondents was 12.8 years, whereas in a study conducted in Rajasthan by Khanna *et al*.(2), the mean age at menarche was found to be 13.2 years.

Unfortunately, 32.5% girls were ignorant about menstruation before menarche in this study. But, each and every girl child should be aware about menstruation, which is an important event at the threshold of adolescence and ideally a mother should be the main informant at this tender age. However, mother was the first informant only in case of 37.5% girls. This gap might be due to poor literacy and socio-economic status of mothers, which have fuelled the inhibitions a mother has to talk to her daughter regarding the significance, hygienic practices and a healthy attitude towards menstruation. The latter will play a long way in maintaining a healthy reproductive tract for each and every girl child who, after she becomes a mother, percolates the healthy message to her female offspring. In a study conducted among 664 schoolgirls aged 14-18 in Mansoura, Egypt by El-Gilany *et al*.(3), mass media were the main source of information about menstrual hygiene, followed by mothers. Another study conducted by Deo *et al*.(1) reported that 40 (42.5%) urban and 41 (55.4%) rural girls were aware about menstruation prior to attainment of menarche. In urban girls, mother was the main source of information about menstruation (27.5%), whereas it was teacher in the rural counterparts (27.01%). Other sources of information were friends, relatives and books. In a study conducted in Rajasthan by Khanna *et al*.(2), nearly 92% of the girls were not aware about the natural phenomenon of menstruation during menarche among women and most of the girls got first information about menstruation from their mothers with other major informants being sisters and friends.

It was observed in this study that 86.25% girls believed it to be a physiological process, whereas in a similar study conducted in Rajasthan by Khanna *et al*.(2), nearly 70% believed that menstruation was not a natural process. It was very sad to observe in the present study that most of the girls did not know about the source of menstrual bleeding and more than half of the girls were ignorant about the use of sanitary pads during menstruation. The above observations might be due to poor literacy level of mothers or absence of proper health education programmes in school, which should focus on menstrual hygiene among girls.

This study shows that majority of the girls preferred cloth pieces rather than sanitary pads as menstrual absorbent. Only 11.25% girls used sanitary pads during menstruation. Apparently, poverty, high cost of disposable sanitary pads and to some extent ignorance dissuaded the study population from using the menstrual absorbents available in the market. In a study conducted in Rajasthan by Khanna *et al*.(2), three-fourths of the girls used old cloth during their periods and only one-fifth reported using readymade sanitary pads. It was observed that the usual practice was to wash the cloth with soap after use and keep it at some secret place till the next menstrual period. To keep the cloth away from prying eyes, these are sometimes hidden in unhygienic places. Privacy for washing, changing or cleaning purpose is something very important for proper menstrual hygiene, but in this study, lack of privacy was an important problem since more than half of the respondents did not possess a covered toilet. Regarding the method of disposal of the used material, most of the girls (73.75%) reused cloth pieces and 57.5% girls properly disposed the used material. In a similar study conducted among 664 schoolgirls aged 14-18 years in Mansoura, Egypt by El-Gilany *et al*.(3), the different aspects of personal hygiene were generally found to be poor, such as not changing pads regularly or at night, and not bathing during menstruation with lack of privacy being an important problem. Different restrictions were practiced by most of the girls in the present study, possibly due to their ignorance and false perceptions regarding menstruation.

## Conclusions

Reproductive tract infections, which has become a silent epidemic that devastates women's life is closely interrelated with poor menstrual hygiene. Therefore, proper menstrual hygiene and correct perceptions and beliefs can protect the womenfolk from this suffering. Before bringing any change in menstrual practices, the girls should be educated about the facts of menstruation, physiological implications, about the significance of menstruation and development of secondary sexual characteristics, and above all, about proper hygienic practices with selection of disposable sanitary menstrual absorbent. This can be achieved through educational television programmes, school nurses/health personnel, compulsory sex education in school curriculum and knowledgeable parents, so that her received education would indirectly wipe away the age-old wrong ideas and make her feel free to discuss menstrual matters including cleaner practices without any hesitation. All mothers irrespective of their educational status should be taught to break their inhibitions about discussing with their daughters regarding menstruation much before the age of menarche.

Lack of privacy is an important problem. In resource poor contexts, where women do not have access to basic facilities such as water, bathroom and privacy, the standard of hygiene one can maintain is severely compromised. There is a need to improve the housing conditions with respect to basic facilities. Universalized use of sanitary pads can be advocated to every girl only by making it available at affordable prices (social marketing).

This study reveals that menstrual hygiene is far from satisfactory among a large proportion of the adolescents while ignorance, false perceptions, unsafe practices regarding menstruation and reluctance of the mother to educate her child are also quite common among them.

Thus, the above findings reinforce the need to encourage safe and hygienic practices among the adolescent girls and bring them out of traditional beliefs, misconceptions and restrictions regarding menstruation.
